# Fish Gelatin-Based Flexible and Self-Healing Hydrogel Modified by Fe_2_(SO_4_)_3_

**DOI:** 10.3390/gels10090557

**Published:** 2024-08-28

**Authors:** Lili Zhang, Haimei Liu, Qin Zhao

**Affiliations:** School of Food Engineering, Ludong University, Yantai 264025, China; 2593@ldu.edu.cn (H.L.); 2800@ldu.edu.cn (Q.Z.)

**Keywords:** fish gelatin, hydrogel, gellan gum, Fe_2_(SO_4_)_3_

## Abstract

The application of fish gelatin (FG) is limited due to its poor mechanical properties and thermal stability, both of which could be significantly improved by gellan gum (GG) found in previous research. However, the FG/GG composite hydrogel was brittle and easily damaged by external forces. It was found that the composite hydrogel with Fe_2_(SO_4_)_3_ showed good flexibility and self-healing properties in the pre-experiment. Thus, the synergistic effect of FG, GG and Fe_2_(SO_4_)_3_ on the mechanical properties of the composite hydrogel was investigated in this study. According to one-way experiments, response surface tests and Texture Profile Analysis, it was found that under the optimum condition of FG concentration 186.443 g/L, GG concentration 8.666 g/L and Fe_2_(SO_4_)_3_ concentration 56.503 g/L, the springiness of the composite cylindrical hydrogel with the height of 25 mm formed in 25 mL beakers (bottom diameter 30 mm) was 7.602 mm. Determination of the rheological properties, compression performance, adhesive performance and self-healing properties showed that the composite hydrogel had good thermal stability, flexibility and self-healing properties with good adhesion, skin compliance and compressive strength, and it was easy to remove. The composite hydrogel showed strong antimicrobial activity against *A. salmonicida* and *V. parahaemolyticus*. All hydrogels showed a uniform and porous structure. The 3D structure of the composite hydrogel was much looser and more porous than the pure FG hydrogel. The flexible and self-healing composite hydrogel with some antimicrobial activity is suitable for the development of medical dressings, which broadens the applications of the composite hydrogel.

## 1. Introduction

Gelatin is a product of partial hydrolysis of collagen [1], containing a large number of functional groups and having the advantages of low antigenicity, strong biodegradability, good biocompatibility, etc. It can be dissolved to form a colloidal solution when solution temperature is higher than its gel point. With the colloidal solution temperature decreasing, the gelatin molecular chains are rearranged, and the triple helix structure is partially restored [2]. Gelatin forms a “denatured” or “structured” gel through intermolecular hydrogen bonding interactions [3]. Recently, the utilization of mammalian gelatin has been restricted due to infectious diseases and religious beliefs. Fish gelatin (FG) is widely available and may be a preferable alternative [4]. However, the mechanical properties of FG-based hydrogel are poorer than that of mammal gelatin, with lower gel points and melting points, which are easy to melt at room temperature (RT, approximately 25 °C). The gel strength, elasticity, and thermal stability are difficult to meet the needs of practical applications, which limits the development of FG. In recent years, a large number of studies have been conducted to improve the stability of gelatin-based hydrogels by modifying gelatin with physical and chemical methods [5].

In a previous study, we found that the gel properties and thermal stability of the tilapia skin gelatin with the concentration of 100 g/L could be significantly improved by gellan gum (GG), with the melting point increasing from 24.3 °C to 57 °C [6]. FG/GG composite hydrogel was stable at RT, and it was a brittle hydrogel. Once the structure was destroyed by external mechanical forces, the mechanical properties and functions of the hydrogel would disappear, shortening the service life of the material. Therefore, it is extremely important to prepare materials with toughness and recoverability to improve their utilization value.

The carboxyl groups in gelatin molecular chains can be used as ligands, and metal ions can form cross-linking points based on metal–ligand coordination bonds with them under certain conditions, thus forming physically enhanced gelatin hydrogels [7]. Metal–ligand bonds are non-covalent interactions with strong bonding strength [8], which are intermediate between covalent bonds and van der Waals forces, with bond energies between 50 and 200 kJ/mol [9]. The introduction of coordination bonds can largely change the mechanical properties of materials, leading to tunable adhesion, toughness and stiffness [10]. There are lots of carboxyl groups in FG molecular chains that can act as ligands and can be formed cross-linking sites based on metal–ligand coordination bonds with metal ions, which can greatly improve the mechanical strength and toughness of FG hydrogels [11], resulting in physically reinforced composite hydrogels [12]. The metal–ligand bonds are easier broken than chemical bonds, which can effectively dissipate energy, obtaining hydrogels with high strength and toughness. Metal coordination can greatly enhance the mechanical properties of various 3D networks by further cross-linking the pre-formed covalent networks [13]. Therefore, physically cross-linked gels with high toughness can be designed based on dynamic metal coordination bonds. Wang et al. [14] immersed the gelatin hydrogels in metal ion solutions, tightening the bonding between the gelatin molecular chains through metal coordination bonds formed by the metal ions with the carboxyl groups in the gelatin, thus enhancing the mechanical properties of the hydrogels.

Most hydrogels are hydrophilic cross-linked 3D systems that are susceptible to external stimuli, which affects the structural integrity of the gel network. Once the structure of the hydrogel is destroyed by external mechanical forces, the mechanical properties and functions of the hydrogel will disappear, shortening the service life of the material. The self-healing property of the hydrogel means that it can repair its structure and restore its mechanical properties after being damaged by external forces. Metal–ligand bonds are reversible and can dissociate and associate rapidly with good energy dissipation properties, and can also recover in a short time [15], realizing rapid repair of the material [16]. Many excellent hydrogel materials with dynamic metal–ligand interactions have been reported in the literature. New hydrogels are fabricated by natural or synthetic polymers with high toughness and self-healing properties based on strong/weak bonding. Strong bonds form the primary structure and control the robustness of the hydrogel, while the breakdown of weak bonds consumes energy, which provides toughness to the hydrogel. The self-healing of the ruptured hydrogels is attributed to the dynamic weak bond recombination. The introduction of metal ions increases the cross-linking density of the gel network, and the formation of dynamically reversible metal–ligand bonds also effectively dissipates a large number of external forces, resulting in the self-healing properties of the hydrogels. The good dynamics of the metal–ligand bonds facilitate energy dissipation and reorganization of the polymer network, resulting in excellent elasticity and self-healing properties of the hydrogel materials. Liang et al. [17] reported the preparation of tough and rapidly recoverable composite hydrogels by soaking the prepared hydrogels in FeCl_3_ solution, conferring the hydrogel’s recoverability and toughness. Soaking gelatin hydrogels in metal ion solutions to form metal–ligand bonds usually take a long time, so we attempted to add metal salts into the composite hydrogel in the pre-experiment. The effect of different high-valent metal salts (Fe_2_(SO_4_)_3_, FeSO_4_, FeCl_2_, FeCl_3_ and CaCl_2_) on the mechanical properties of the composite hydrogel was investigated, finding that the composite hydrogel with Fe_2_(SO_4_)_3_ showed good flexibility and self-healing properties.

The mechanical properties of hydrogels are generally characterized by Young’s modulus. The proportional relationship between stress and strain in the process of elastic deformation of materials is the Young’s modulus, also named elastic modulus, which is the main mechanical characteristic parameter. The larger the elastic modulus, the less likely the material is to undergo deformation. At present, the methods for measuring the elastic modulus of hydrogels are not fixed, and they mainly include the tensile method, compression method, and indentation method. Texture Profile Analysis (TPA) can be used to measure the hardness, elasticity, resilience and cohesiveness of hydrogels by twice compressing the sample.

Natural FG hydrogel is limited in the application of demanding high mechanical properties due to its low elasticity and limited self-healing ability. The purpose of this study was to develop a thermal, stable FG-based hydrogel with good flexibility and certain self-healing properties so as to expand its application in the field of biomaterials. Therefore, the synergistic effect of FG, GG and Fe_2_(SO_4_)_3_ on the mechanical properties of the composite hydrogel was investigated through one-way experiments and response surface tests. Then the changes in spatial reticulation of the gel structures were elucidated. In addition, gelatin is susceptible to the growth of bacteria and microorganisms, which limits its application. The antibacterial and adhesion properties of the composite hydrogels were also investigated.

## 2. Results and Discussion

### 2.1. Results of One-Way Test for the Preparation of Fish Gelatin-Based Flexible Hydrogel

**Figure 1:**
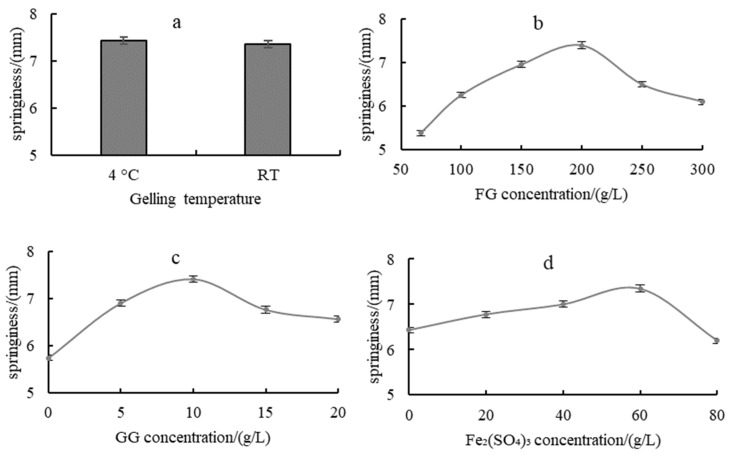
Effects of gelling temperature (**a**), FG concentration (**b**), GG concentration (**c**) and Fe_2_(SO_4_)_3_ concentration (**d**) on the springiness of fish gelatin-based flexible hydrogel.

The effects of gelling temperature on the springiness of the composite hydrogels Figure 1a show no significant changes. Hydrogels were gelled at RT in the following experiments. Figure 1b–d show the effects of FG concentration, GG concentration and Fe_2_(SO_4_)_3_ concentration on the springiness of the composite hydrogels, respectively. All curves showed a trend of first increasing and then decreasing, with different changing rates. The optimal values for FG concentration, GG concentration and Fe_2_(SO_4_)_3_ concentration are 200 g/L, 10 g/L and 60 g/L, respectively.

The composite hydrogel with moderate addition of Fe_2_(SO_4_)_3_ showed good springiness. With too little addition, the hydrogel was viscous, and with too much addition, the hydrogel was hard, both of which were poor in springiness. 

### 2.2. Response Surface Optimization ANOVA for the Preparation of Fish Gelatin-Based Flexible Hydrogel

#### 2.2.1. Response Surface Test Design and Results

According to the results obtained above, three factors, namely, FG concentration (A), GG concentration (B) and Fe_2_(SO_4_)_3_ concentration (C), were taken as independent variables and optimized by response surface design to obtain the optimum process for the preparation of fish gelatin-based flexible hydrogel. A total of 17 hydrogels were produced and measured, with the scheme and results shown in Table 1.

#### 2.2.2. Establishment and Analysis of Response Surface Regression Model

The data in Table 1 were analyzed by Design Expert 12 to derive the regression model equation for the springiness of fish gelatin-based flexible hydrogel (Y) versus FG concentration (A), GG concentration (B) and Fe_2_(SO_4_)_3_ concentration (C) as follows:

Y = 7.55 − 0.1687A − 0.155B − 0.1138C + 0.08AB + 0.1875AC + 0.19BC − 0.4103A^2^ − 0.3928B^2^ − 0.6152C^2^.

The correctness of the regression equation was tested by ANOVA.

It can be seen from Table 2 that the F-value of the model was 32.87 and the *p*-value was less than 0.001, indicating that the model was highly significant. The *p*-value of the lack-of-fit term was 0.9716 > 0.05, indicating that the effect of the extra-experimental factors was not significant. The Predicted R^2^ of 0.9466 is in reasonable agreement with the Adjusted R^2^ of 0.9472, indicating that this model was highly credible. A, B and C had significant effects on the springiness of the flexible hydrogel (*p* < 0.05), among which A and B reached highly significant levels (*p* < 0.01). The secondary terms A^2^, B^2^ and C^2^ reached highly significant levels (*p* < 0.01), and AC and BC reached significant levels (*p* < 0.05). The influence order of each factor was A > B > C, i.e., FG concentration > GG concentration > Fe_2_(SO_4_)_3_ concentration.

#### 2.2.3. Two-Factor Interaction Analysis

**Figure 2:**
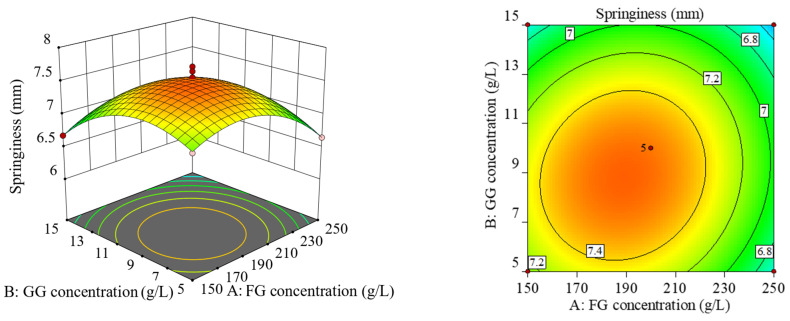

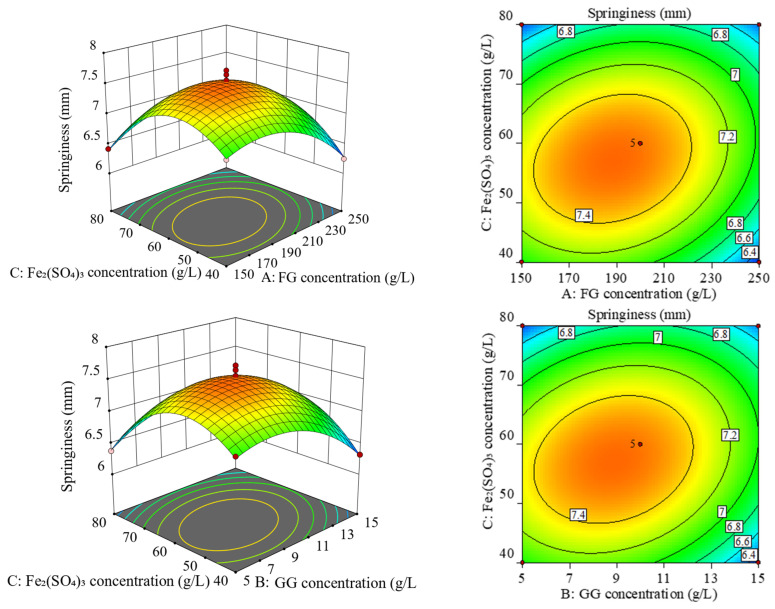
Response surface plot and contour plot of the interaction of various factors.

The projection of the response surface graph in the horizontal direction is a contour line. The elliptical contour line indicates a highly significant interaction between these two factors, while the circular contour indicates no significant interaction. As can be seen from Figure 2, the response surface of factor (B) and factor (C) was the steepest, and the contour was elliptical, indicating their significant interaction effect. The response surface of factor (A) and factor (C) was the second steepest, and the contour was the same as that of factor (B) and factor (C), indicating their significant interaction effect. The slopes of the response surface of factor (A) and factor (B) were the smallest, and their interaction effect was not significant.

#### 2.2.4. Determination of Optimal Conditions and Verification of Regression Model

The optimal process conditions of FG concentration 186.443 g/L, GG concentration 8.666 g/L and Fe_2_(SO_4_)_3_ concentration 56.503 g/L were obtained by Design Expert 12 software. Under the corresponding conditions, the springiness of fish gelatin-based flexible hydrogel was predicted at 7.602 mm. Based on the operability and convenience of industrial production, FG concentration, GG concentration and Fe_2_(SO_4_)_3_ concentration were adjusted to 186 g/L, 9 g/L and 57 g/L, respectively. To verify the operability of the model, three parallel experiments were conducted under the above conditions, and the springiness was 7.58 ± 0.12 mm. Compared with the predicted springiness value in the model, the error was within 1%, indicating that the model was credible. 

As mentioned above, many researchers soaked FG hydrogel in a metal ion solution to change its gel properties, which usually took a long time. In this study, metal ions were added into the system to improve the flexibility of the hydrogel, which simplified the preparation process and made it easy to realize in industrial production.

### 2.3. Rheological Properties of the Composite Hydrogel

Complex modulus G refers to the energy used to resist deformation. When the material undergoes deformation, the larger the complex modulus, the stronger the ability of the material to resist deformation. G = G′+ i G. G′, known as storage modulus or elastic modulus, is the energy stored in the material when deformation occurs due to elastic (reversible) deformation, reflecting the material’s elasticity. G″, known as loss modulus or viscous modulus, refers to the energy lost due to viscous deformation (irreversible) when the material undergoes deformation, reflecting the material’s viscosity. 

**Figure 3:**
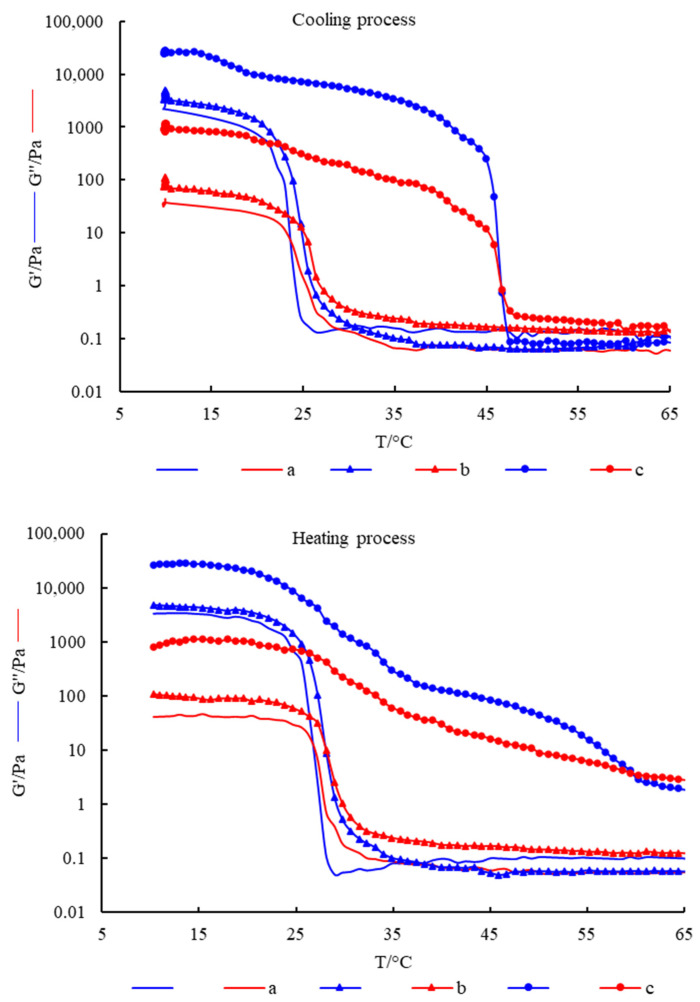
The changes of G′ and G″ of hydrogels with temperature. (**a**) FG, (**b**) FG + Fe_2_(SO_4_)_3_ and (**c**) FG + GG + Fe_2_(SO_4_)_3_.

Figure 3 shows the changes of G′ and G″ of different hydrogels with temperature during the cooling process and heating process. The results show that, in the cooling process, both G′ and G″ of the hydrogels gradually increased with the decrease of temperature. When the temperature dropped to a certain point, there was an intersection of G′ and G″, which indicated that the elastic component in the system increased, and the hydrogel started to gel. As the temperature continued to decrease, G′ was always greater than G″. In the heating process, G′ and G″ of the hydrogel gradually decreased with the increase in temperature. When the temperature rose to a certain point, the intersection point of G′ and G″ occurred, which indicated that the viscous component in the system increased, and the hydrogel began to melt. With the continuous increase of temperature, G″ was always greater than G′. The above results indicated that the hydrogels were all thermoreversible gels.

In the temperature scanning test, the intersection of G′ and G″ was usually used as the turning point of gel or melt in dynamic viscoelastic analysis. Cho et al. [18] determined the gel point and melting point of gelatin by temperature scanning. Therefore, the intersection of G′ and G″ in the cooling process in Figure 3 was the gel point, and the intersection of G′ and G″ in the heating process was the melting point. It can be seen from the figure that the gel point of pure fish gelatin hydrogel was about 24 °C, and the melting point was about 27 °C, which was not conducive to its gelation and preservation at RT. The addition of Fe_2_(SO_4_)_3_ improved the gel point and melting point slightly but still could not meet its processing and application at RT. The simultaneous addition of Fe_2_(SO_4_)_3_ and gellan gum significantly increased the gel point and melting point to 45 °C and 60 °C, respectively. Therefore, the composite hydrogel can be formed at RT, and the gel properties are stable, which not only saves production costs but also broadens the use conditions of hydrogel, which is of great significance in broadening the application of fish gelatin. 

### 2.4. Compression Performance, Adhesive Performance and Self-Healing Properties of the Composite Hydrogel

**Figure 4:**
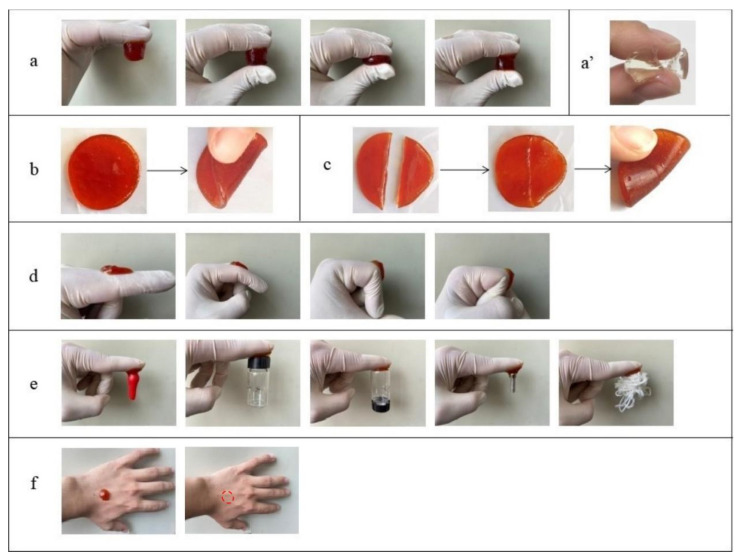
Digital photos of the composite hydrogel under (**a**,**a′**) compression, (**b**) folding, (**c**) self-healing, (**d**) bending, (**e**) adhering to different objects and (**f**) attachment to the skin and after removal (Red dotted circle was the location where the composite hydrogel attached). (**a**–**f**) FG + GG + Fe_2_(SO_4_)_3_ and (**a′**) FG.

The pure FG hydrogel was brittle with weak mechanical properties and no self-healing property (Figure 4a′), while the composite hydrogel was good in compression and folding performance, with excellent self-healing performance (Figure 4a,b). No fracture occurred when the hydrogels were compressed or folded, indicating their toughness. 

To pure FG, the gelatin molecules exhibit a random helical structure, and a large number of water molecules can be bound between the molecular chains above the melting point. When below the gel point, some segments of the molecule rotate into a left-handed helix, and three neighboring left-handed helical segments refold through hydrogen bonding to form a helical linkage region similar to that of natural collagen, which becomes a cross-linking point [19]. Other cross-linking points form nearby, creating a 3D network, which in turn forms FG hydrogel. The cross-links between the polymer chains were not strong enough. Metal ions can form metal–ligand coordination bonds with appropriate ligands under certain conditions. In this study, Fe^3+^ was introduced for coordination cross-linking to enhance the mechanical properties of the hydrogels. The Fe^3+^ cross-linked hydrogels were found to be highly flexible. Fe^3+^ occupied the position of the original water molecules attached to the carboxyl groups of the side chains of FG molecules through coordination and formed strong ionic cross-links between molecular chains. The helical linkage regions and cross-linking sites of metal ions in FG hydrogels gradually increased with temperature decreasing. In addition, Fe^3+^ was able to react with three carboxylate ions from different molecular chains and form a compact 3D linkage structure so that the chains of the FG hydrogel are tightly linked together, thus making the structure of the hydrogel more compact, and obtaining ionically cross-linked hydrogel with high strength and high elasticity. The strength of the interaction between metal ions and the carboxyl groups in FG molecule determined its mechanical strength. Lin et al. [20] developed a tough hydrogel by further cross-linking a chemically cross-linked network containing acrylic acid components with Fe^3+^.

In Figure 4c, the hydrogels cut into two segments were spliced together, and the free Fe^3+^ diffused into the interface to interact with the carboxyl groups of the FG chains without any external interference, showing excellent self-healing properties. Fe^3+^ cross-linked hydrogels can dissipate most of the energy exerted on the hydrogel during force deformation by dynamically reversible metal–ligand bonds, which endows the hydrogel with good self-healing properties. Therefore, hydrogels can restore their original structures and properties after being damaged, thus increasing the reliability and safety in realizing their functions [21].

The adhesion properties of the composite hydrogel are shown in Figure 4d,e. It was found that the composite hydrogel had good adhesion, skin compliance and compressive strength, and it was easy to remove. The sheet hydrogel firmly adhered to the joint. The hydrogel maintained good adhesion even when the finger joints flexed at 0°, 45°, 90° and 135°. The hydrogels had excellent adhesive properties on various substrates (including rubber, plastic, glass, stainless steel, leather, etc.) [22], having potential applications in the field of wound dressings [23].

Hydrogel as a wound dressing should be adhesive and easy to remove from the wound site to avoid secondary injury. Figure 4f shows that when the hydrogel was peeled off from the skin, no residue was observed on the skin. The hydrogel adhering to the skin was easily removed, indicating that the hydrogel can be used in the study of wound dressing materials.

### 2.5. Antibacterial Activity of Hydrogels

Gelatin as a protein is easy to harbor bacteria and microorganisms, so the antimicrobial properties of pure gelatin hydrogels are poor, which limits their applications. Metal ions usually have broad-spectrum antimicrobial properties and are often used to prepare antimicrobial biomaterials. 

**Figure 5:**
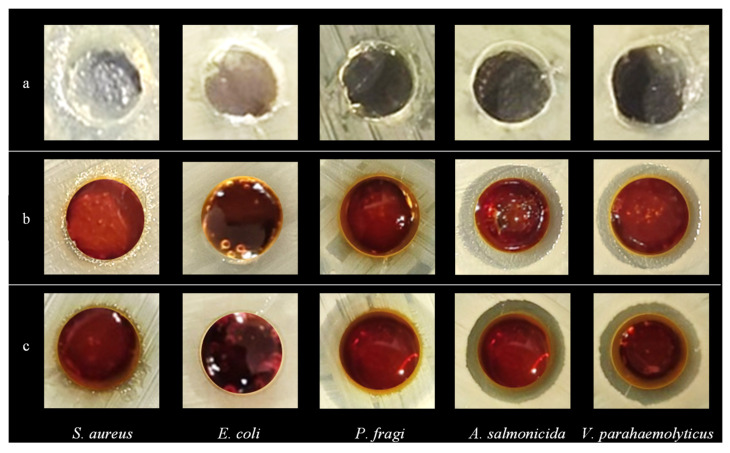
Images of the inhibition zone of hydrogels. (**a**) FG, (**b**) FG + Fe_2_(SO_4_)_3_ and (**c**) FG + GG + Fe_2_(SO_4_)_3_.

**Figure 6:**
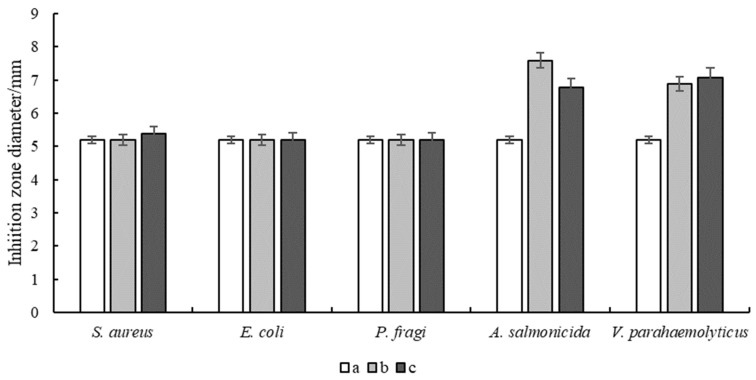
Inhibition zone diameter of hydrogels. (**a**) FG, (**b**) FG + Fe_2_(SO_4_)_3_ and (**c**) FG + GG + Fe_2_(SO_4_)_3_.

Antimicrobial experiments of the hydrogels were carried out to investigate their inhibitory effects on *E. coli*, *S. aureus*, *P. fragi*, *A. salmonicida* and *V. parahaemolyticus* (Figure 5 and Figure 6). The results showed that the pure FG hydrogel had no antimicrobial effect on the above strains. The composite hydrogel showed strong antimicrobial activity against *A. salmonicida* and *V. parahaemolyticus* with obvious inhibitory circles in the medium. Hydrogels with bioadhesive properties can be used as wound dressings to seamlessly adhere to the wound site for a long period of time, providing a temporary barrier against external bacterial infections and preventing secondary damage from friction with the wound surface. *A. salmonicida* is an important pathogen of fish and it can be inhibited by the composite hydrogel, which can be made into films for fish preservation. *V. parahaemolyticus* is a kind of marine bacterium mainly from fish, shrimps, crabs, shellfish, seaweeds, etc. It is harmful to the human body through wound infection, which can be significantly inhibited by the composite hydrogel. Therefore, it is of great significance to develop it as a wound dressing to protect the wounds of consumers, especially fishers.

The antibacterial properties of hydrogel might be related to the introduction of Fe^3+^, which could interact with the phospholipid bilayer in the bacterial cell membrane or produce oxidation reactions with enzymes, proteins, etc., in the cell to destroy its biological activity and cause bacterial death.

### 2.6. FTIR Spectra of Hydrogels

**Figure 7:**
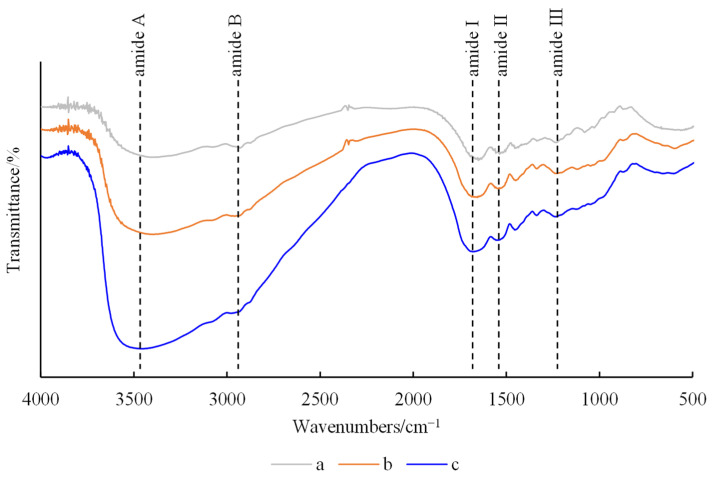
FTIR spectra of hydrogels. (**a**) FG, (**b**) FG + Fe_2_(SO_4_)_3_ and (**c**) FG + GG + Fe_2_(SO_4_)_3_.

The functional groups and intermolecular interactions in hydrogels can be determined by FT-IR spectral analyses. The FTIR spectra of the hydrogels are shown in Figure 7. 

All hydrogels showed characteristic peaks of amide A, amide B, amide I, amide II and amide III, with similar peak shapes and bands, albeit with slight changes in peak positions. All hydrogels had strong, broad absorption peaks at 3200–3500 cm^−1^, corresponding to the tensile vibration of O-H and N-H [24]. The peak at 2927 cm^−1^ represented the symmetric stretching vibration of C-H and -NH_2_, indicating the presence of aman ide B band. The amide I peak of FG hydrogel was located at 1643 cm^−1^, related to C-O and C-N stretching, and was very sensitive to changes in protein secondary structure [25]. The peak intensity of the amide I band of the composite hydrogels was enhanced, and the peak position moved to a higher wavenumber, indicating their more orderly internal structure. The amide II peak of FG hydrogel is located at 1519 cm^−1^, which can be attributed to N-H bending mode and C-N stretching vibration [26]. The amide II band of the composite hydrogels slightly moved to a higher wavenumber, indicating that Fe^3+^ may interact with FG’s carboxyl group (-COO-). The amide III peak of FG hydrogel was located at 1226 cm^−1^, which is attributed to C-N stretching and N-H bending of the peptide bond.

It can be seen from the FTIR results that Fe^3+^ has a certain impact on the secondary structure of FG, leading to a more orderly structure in the composite hydrogels. It was speculated that the toughness of the composite hydrogels was enhanced by the metal coordination bond formed between carboxyl groups in the gelatin molecular chain and Fe^3+^ [27]. The peak intensity and position of the two composite hydrogels were similar, indicating that the addition of GG has no significant effect on the characteristic bands of the hydrogel. It was speculated that the network structure of the hydrogel was mainly the result of the interaction between FG molecule and Fe^3+^, while GG was only filled into the reticulation, making the three-dimensional structure of the hydrogel more compact.

### 2.7. SEM of Hydrogels

**Figure 8:**
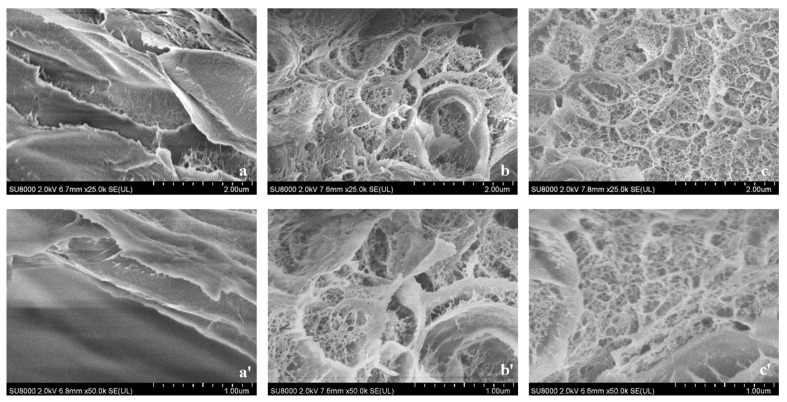
SEM images ((**a**–**c**) ×25,000, (**a′**–**c′**) ×50,000) of hydrogels. (**a**,**a′**) FG, (**b**,**b′**) FG + Fe_2_(SO_4_)_3_ and (**c**,**c′**) FG + GG + Fe_2_(SO_4_)_3_.

In order to explore the internal microstructure, the cross-sectional micromorphology of the hydrogels was observed by SEM, including the apparent morphology and compactness (Figure 8). A typical uniformly loose and porous network structure with thick pore walls formed in the pure FG hydrogel, showing a tight overall structure. When Fe^3+^ was added, the -COO- in the molecular chains complexed with Fe^3+^, leading to the cross-linking between the molecular chains inside the hydrogel, forming a more compact 3D network structure with richer and denser pores. The pore diameters became smaller, and the pore walls became thinner. The spatial structure of the hydrogel with GG had almost no change with a more uniform microstructure. It was hypothesized that GG was dispersed in the gelatin solution, playing the filling effect, but it did not affect the molecular structure of FG.

All hydrogels showed a uniform and porous structure conducive to the exchange of nutrients [28], which is suitable for the development of medical dressings.

## 3. Conclusions

The pure FG hydrogel is a brittle gel with poor mechanical properties [29] and no self-healing properties or antimicrobial activity. The composite hydrogel showed good flexibility and self-healing properties with good adhesion, skin compliance and compressive strength, and it was easy to remove. It showed strong antimicrobial activity against *A. salmonicida* and *V. parahaemolyticus.* The 3D structure of the composite hydrogel was much looser and more porous than the pure FG hydrogel. The preparation process of the composite hydrogel obtained was simplified that is easy to realize in industry. The self-healing hydrogel with some antimicrobial activity is suitable for the development of medical dressings, which broadens its applications in the field of biomaterials.

## 4. Materials and Methods

### 4.1. Materials and Reagents

Tilapia skin gelatin (frozen force 200, food-grade) was purchased from Xinxi Biotechnology Co., Ltd., Shanghai, China. Low-acyl GG (food-grade) was purchased from Wangxin Biotechnology Co., Ltd., Shanghai, China. The other chemical reagents were of analytical grade. Fe_2_(SO_4_)_3_ was purchased from Sinopharm Chemical Reagent Co., Shanghai, China.

### 4.2. Preparation of Hydrogels

The composite hydrogels were prepared by one-pot synthesis [30]. A certain amount of tilapia skin gelatin, Fe_2_(SO_4_)_3_, and low acyl GG were dissolved under stirring in deionized water at 65 °C. The homogenous solution was placed in 25 mL beakers (bottom diameter 30 mm) with a height of 25 mm and formed hydrogels at 4 °C in the refrigerator or RT for 16–18 h to be tested. 

The pure FG hydrogel was prepared in similar conditions based solely on the above composite gel and placed at 4 °C in the refrigerator for 16–18 h before testing. 

### 4.3. Determination of the Springiness of the Hydrogels

Hydrogels were placed at RT for 2 h, and then the textural properties were determined by texture analyzer (CT3-10K texturing apparatus, Brookfield Corporation, New York, NY, USA) referred to Sow et al. [31] with modifications. TPA mode was selected for measurement, using a TA5 probe (diameter 12.7 mm, cylindrical probe). The pre-test speed, test speed, return speed and trigger force were 1.00 mm/s, 2.00 mm/s, 2.00 mm/s and 5 g, respectively. The target mode was selected as 30% deformation. The springiness was measured three times in each group, and the average value was taken.

### 4.4. One-Way Experimental Design for the Preparation of Fish Gelatin-Based Flexible Hydrogel

#### 4.4.1. Effect of Gelling Temperature on the Springiness of Fish Gelatin-Based Flexible Hydrogel

The effect of different gelling temperatures, i.e., 4 °C, RT, on the springiness of fish gelatin-based flexible hydrogel was investigated with the FG concentration 20 g/L, GG concentration 10 g/L and Fe_2_(SO_4_)_3_ concentration 60 g/L.

#### 4.4.2. Effect of FG Concentration on the Springiness of Fish Gelatin-Based Flexible Hydrogel

The effect of different FG concentrations, i.e., 66.7 g/L, 100 g/L, 150 g/L, 200 g/L, 250 g/L, 300 g/L, on the springiness of fish gelatin-based flexible hydrogel was investigated with the GG concentration 10 g/L, Fe_2_(SO_4_)_3_ concentration 60 g/L and gelling temperature RT.

#### 4.4.3. Effect of GG Concentration on the Springiness of Fish Gelatin-Based Flexible Hydrogel

The effect of different GG concentrations, i.e., 0, 5 g/L, 10 g/L, 15 g/L, 20 g/L, on the springiness of fish gelatin-based flexible hydrogel was investigated with the FG concentration 20 g/L, Fe_2_(SO_4_)_3_ concentration 60 g/L, and gelling temperature RT.

#### 4.4.4. Effect of Fe_2_(SO_4_)_3_ Concentration on the Springiness of Fish Gelatin-Based Flexible Hydrogel

The effect of different Fe_2_(SO_4_)_3_ concentrations, i.e., 0, 20 g/L, 40 g/L, 60 g/L, 80 g/L, on the springiness of fish gelatin-based flexible hydrogel was investigated with the FG concentration 20 g/L, GG concentration 10 g/L, and gelling temperature RT.

### 4.5. Response Surface Test for the Preparation of Fish Gelatin-Based Flexible Hydrogel

Based on the results of the one-way test, three factors, namely, FG concentration, GG concentration, and Fe_2_(SO_4_)_3_ concentration, were selected for the response surface experimental design, and according to Box–Behnken design, the methods are shown in Table 3.

### 4.6. Determination of Rheological Properties

The dynamic viscoelasticity of the hydrogels was measured by rheometer (AR1000, TA Instruments, Inc., New Castle, DE, USA), according to Zhang et al. [6], with some modifications. The parallel plate clamp with a diameter of 40 mm was used, and the distance between the upper and lower plates was 1 mm. The scanning frequency was 1 Hz, and the stress was 3.0 Pa. The temperature scanning program was set as follows: the initial temperature was 65 °C, which decreased to 10 °C at the rate of 2 °C/min, and then raised to 65 °C at the same rate after 5 min of stabilization. The first intersection point of storage modulus G′ and loss modulus G″ during the cooling and heating process was fish gelatin’s gel point and melting point, respectively.

### 4.7. Compression Performance, Adhesive Performance and Self-Healing Properties

The prepared hydrogels were compressed, cut into two pieces and adhered to the surfaces of different materials to observe the compression performance, adhesive performance [32], and self-healing properties, recorded with a digital camera to visually express the adhesive performance of hydrogels. 

### 4.8. Antibacterial Activity Assay

The antimicrobial properties of the hydrogels were tested against the Gram-negative bacterium Escherichia coli (*E. coli*) and the Gram-positive bacterium Staphylococcus aureus (*S. aureus*), Pseudomonas fragi (*P. fragi*), Aeromonas salmonicida (*A. salmonicida*) and Vibrio parahaemolyticus (*V. parahaemolyticus*), referred to Lin et al. [33] with modifications. They were incubated at 37 °C for 24 h with shaking, obtaining a bacterial suspension with a certain concentration. The bacterial suspension was diluted to the concentration of 10^6^~10^7^ CFU/mL with sterile PBS buffer, obtaining the bacterial suspension used in the experiment. The hydrogels were made into discs with a thickness of 2 mm and a diameter of 5 mm, sterilized by irradiation under UV light for 30 min. An amount of 100 μL bacterial suspension was placed in a sterilized LB solid culture medium and evenly coated with an applicator. After inoculation, two holes with a diameter of 10 mm were punched into each culture medium, and the prepared gel samples were put into the holes. The inverted culture dishes were incubated continuously for 24 h at 37 °C in a constant temperature and humidity incubator to observe the bacterial growth. The bacterial inhibition effects of the hydrogels were evaluated according to the size of the inhibition circle formed around the hydrogel slices.

### 4.9. Analyses of Fourier Transform Infrared (FT-IR) Spectra

The FT-IR spectra of freeze-dried hydrogels were recorded by an FT-IR spectrophotometer (IRTracer-100, Shimadzu Corporation, Kyoto, Japan) [34]. Dried samples were ground with KBr and then made into pellets. The FT-IR spectra were measured over a wavenumber range from 400 to 4000 cm^−1^, with 64 scans and a resolution of 4 cm^−1^. KBr without samples was scanned as a baseline. PeakFit v 4.12 software was used for baseline correction.

### 4.10. Scanning Electron Microscopy (SEM)

The hydrogels were rapidly frozen in liquid nitrogen and lyophilized for 24 h. Then, a small piece of each brittle sample was taken and stuck to the sample stage and then placed in an ion sputtering apparatus in a vacuum environment with sprayed platinum for 30 s, observed by Scanning Electron Microscope (SU8010, Hitachi Company, Tokyo, Japan) [34]. Acceleration voltage was imaged at 5.0 KV, and all images were digitally obtained by applying the XT microscope control software (version 6.28).

### 4.11. Statistical Analysis

Microsoft Excel 2016 software was Used to analyze the average of single-factor experimental data. Statistical analysis was performed using variance analysis (ANOVA). The results were expressed as mean ± SD, and the differences were considered to be statistically significant at *p* < 0.05.

Design Expert 12 was used to perform a graphical analysis of the experimental results of response surface analysis.

## Figures and Tables

**Table 1:** Response surface design and results.

**Number**	**A.** **FG Concentration** **/(g/L)**	**B.** **GG Concentration** **/(g/L)**	**C.** **Fe_2_(SO_4_)_3_ Concentration** **/(g/L)**	**Y.** **Springiness** **/(mm)**
1	150	5	60	7.13
2	250	5	60	6.65
3	150	15	60	6.68
4	250	15	60	6.52
5	150	10	40	6.98
6	250	10	40	6.25
7	150	10	80	6.42
8	250	10	80	6.44
9	200	5	40	7.03
10	200	15	40	6.32
11	200	5	80	6.38
12	200	15	80	6.43
13	200	10	60	7.72
14	200	10	60	7.65
15	200	10	60	7.33
16	200	10	60	7.56
17	200	10	60	7.48

**Table 2:** Variance analysis of response surface results.

Source	Sum of Squares	df	Mean Square	F-Value	*p*-Value	Significance
Model	4.11	9	0.4569	32.87	<0.0001	**
A	0.2278	1	0.2278	16.39	0.0049	**
B	0.1922	1	0.1922	13.83	0.0075	**
C	0.1035	1	0.1035	7.45	0.0294	*
AB	0.0256	1	0.0256	1.84	0.2169	not significant
AC	0.1406	1	0.1406	10.12	0.0155	*
BC	0.1444	1	0.1444	10.39	0.0146	*
A^2^	0.7087	1	0.7087	50.98	0.0002	**
B^2^	0.6495	1	0.6495	46.72	0.0002	**
C^2^	1.59	1	1.59	114.66	<0.0001	**
Residual	0.0973	7	0.0139			
Lack of Fit	0.005	3	0.0017	0.0726	0.9716	not significant
Pure Error	0.0923	4	0.0231			
Cor Total	4.21	16				

Note: * Represented significant differences between the data (* *p* < 0.05, ** *p* < 0.01).

**Table 3:** The levels of different factors in response surface test.

Level	A. FG Concentration /(g/L)	B. GG Concentration /(g/L)	C. Fe_2_(SO_4_)_3_ Concentration /(g/L)
1	150	5	40
2	200	10	60
3	250	15	80

## Data Availability

All data and materials are available on request from the corresponding author. The data are not publicly available due to ongoing research using a part of the data.
